# Allergic bronchopulmonary mycosis caused by *Scedosporium apiospermum*: A case report

**DOI:** 10.1016/j.rmcr.2024.102092

**Published:** 2024-08-22

**Authors:** Ryota Okazaki, Shino Arita, Hinako Hoshio, Naoki Uetani, Genki Inui, Hiroki Ishikawa, Takafumi Nonaka, Hiroki Kohno, Yoshihiro Funaki, Tomoya Harada, Masato Morita, Akira Watanabe, Akira Yamasaki

**Affiliations:** a Division of Respiratory Medicine and Rheumatology, Faculty of Medicine, Tottori University, Tottori, Japan; b Medical Mycology Research Center, Chiba University, Chiba, Japan

**Keywords:** Allergic bronchopulmonary mycosis, *Scedosporium apiospermum*, Eosinophil, Mucous plugs, Antifungal drug

## Abstract

A 61-year-old woman, hospitalized for a persistent cough and dyspnea, had no history of bronchial asthma, but was undergoing chemotherapy for methotrexate-related lymphoproliferative disorder due to rheumatoid arthritis. Her peripheral blood eosinophil count was significantly increased, and chest CT revealed left lower lobe atelectasis and high-attenuation mucus. Bronchoscopy revealed mucous plugs and pathological examination revealed numerous eosinophils and filamentous fungi. Allergic bronchopulmonary mycosis (ABPM) caused by *Scedosporium apiospermum* was diagnosed using culture and genetic analyses. Treatment with corticosteroids and antifungal drugs led to improvement. ABPM caused by *S. apiospermum* is extremely rare, emphasizing the importance of species identification.

## Highlights

• ABPM caused by rare fungal infections, including those of the *S. apiospermum*, may occur in patients without asthma or with low serum IgE levels.
• The new clinical diagnostic criteria for ABPM in Japan now allow for the diagnosis of ABPM even in cases without asthma.
• Owing to variations in antifungal susceptibility among fungal species, it is important to identify the fungal species whenever possible.

## 1 Introduction

Allergic bronchopulmonary mycosis (ABPM) is a chronic respiratory disease where saprophytic fungi (molds), primarily *Aspergillus* spp. grow in the airways of adult patients with asthma or cystic fibrosis, inducing type I and III allergic reactions. Although *Aspergillus* spp. are often the causative fungi, data on the causative fungal species of non-*Aspergillus* ABPM are limited.

Diagnostic criteria for allergic bronchopulmonary aspergillosis (ABPA) by Rosenberg et al. [1] and the International Society for Human and Animal Mycology (ISHAM) [2] were used. New clinical diagnostic criteria for ABPM have been proposed in Japan, allowing diagnosis even in cases without asthma or with non-*Aspergillus* ABPM [3].

*Scedosporium apiospermum,* a filamentous fungus found in dead organisms [4], can cause various infections, including pneumonia [5]. Reports of *S. apiospermum* causing ABPM are rare, but we report a case here. Genetic analysis identified the fungus, and the patient showed improvement with corticosteroids and antifungal drugs.

## 2 Case presentation

A 61-year-old female treated for rheumatoid arthritis since age 56 developed methotrexate-associated lymphoproliferative disorder (MTX-LPD) at 58. Despite MTX discontinuation, LPD recurred at age 61. She underwent bendamustine-rituximab (BR) combination therapy, resulting in LPD reduction. However, after three BR courses, she developed cough and sputum leading to referral to the respiratory medicine. Blood tests revealed peripheral blood eosinophilia, and chest computed tomography (CT) revealed left lower lobe atelectasis and a high-attenuation mucus (HAM) plug in the left lower lobe bronchus. She was admitted to the hospital with suspected ABPM.

**Fig. 1:**
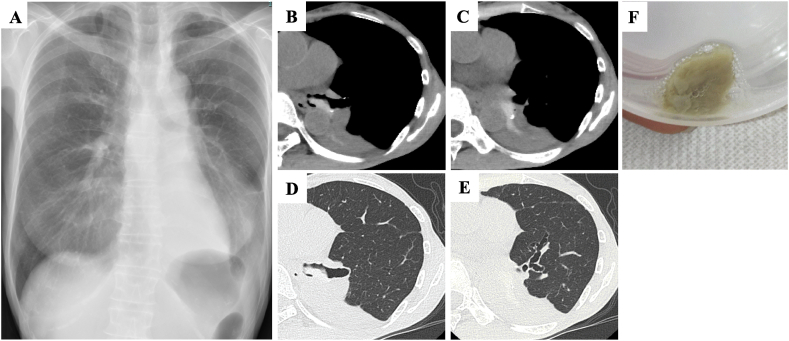
Chest imaging findings on admission. Chest radiograph showing the collapse of the left lower lung (A). High-resolution CT showing high-attenuation mucus (HAM) in the lower lobe bronchus (B, C) and left lobe atelectasis (D, E). Mucus plugs in the sputum (F).

**Fig. 2:**
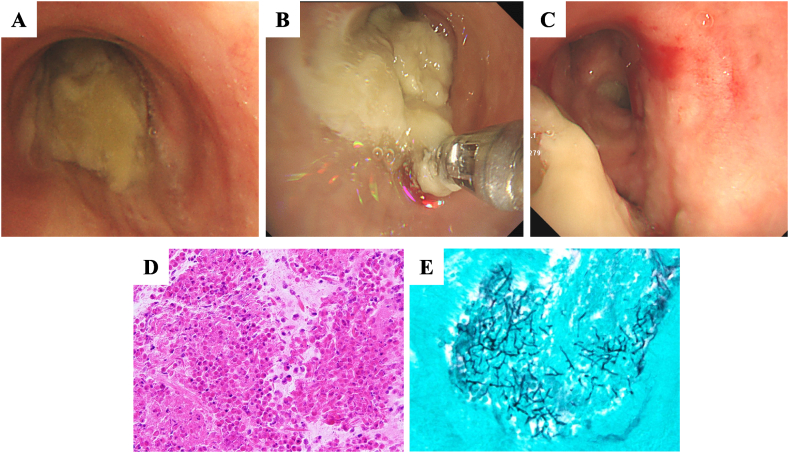
Bronchoscopic findings showing a yellowish-white mucus plug in the left main bronchus (A). The plugs were very viscous, making it difficult to remove all of them with suction and forceps using bronchoscopy (B). The mucus plugs remained in the left inferior lobe branch due to complications of airway mucosal edema and decreased SpO_2_ (C). Histopathological findings for mucus plugs using haematoxylin and eosin staining. The specimen shows a cluster of eosinophils and Charcot–Leyden crystals (D). Histopathological findings of the mucus plug using Grocott's stain. The specimen shows mycelia (E).

The patient, a non-smoker with no history of bronchial asthma or allergic rhinitis presented with her vital signs: temperature 36.8 °C, pulse 101 beats/min, blood pressure 105/67 mmHg, and oxygen saturation (SpO_2_) 92 % on room air. She was thin, 156.0 cm tall, weighed 33.8 kg, with a BMI of 13.8. Auscultation showed absent breath sounds with decreased sounds in the lower left lung. Laboratory results revealed leukocytosis with eosinophilia (10,700/μL; eosinophils 5018/μL) while IgE levels were normal (<20 IU/mL) and specific IgE against Aspergillus was negative. Chest imaging revealed decreased opacity in the left lower lung field (Fig. 1A) with CT showing atelectasis in the left lower lobe and high-attenuation mucus (HAM) in the left lower lobe bronchus (Fig. 1B–E). Pulmonary function tests indicated, restrictive ventilatory impairment and forced expiratory volume in 1 second (FEV1) of 1.16 L (66.2 % predicted), forced vital capacity (FVC) of 1.66 L (60.1 % predicted), and an FEV1/FVC ratio of 66.2 %. Fractional exhaled nitric oxide (FeNO) levels were elevated at 194 ppb (normal range, <37 ppb). The expectorated mucus plug revealed abundant eosinophils with a positive culture for *Scedosporium* sp. (Fig. 1F), which was genetically identified as *S. apiospermum* at Chiba University Medical Mycology Research Center. Bronchoscopy performed on the second day of hospitalization revealed mucus plugs in the left main bronchus, hindering visualization of the left secondary bronchus (Fig. 2A). Attempts to remove the plugs using suction and forceps (Fig. 2B) was partly successful, but residual plugs remained due to airway mucosal edema, causing decreased SpO_2_ (Fig. 2C), and methylprednisolone (mPSL) 125 mg was administered. Pathological examination revealed numerous eosinophils (Fig. 2D) and positive filamentous fungi staining in the plugs (Fig. 2E). The genetic analysis of the pathological specimens revealed the fungus as *S. apiospermum,* confirming the mucus plugs caused by the fungus. Diseases such as bronchial asthma and eosinophilic pneumonia were considered due to the patient's cough and peripheral blood eosinophilia. However, the patient had no asthma history, and the mucus plugs in the central airways strongly suggested ABPM. Lung cancer and bronchial atresia were ruled out, as chest CT scans during LPD treatment showed no signs of lung cancer. Mycobacterial infection was also excluded due to negative sputum tests and negative anti-Mycobacterium avium complex antibodies and T-SPOT. TB test. ABPA was considered, but Aspergillus antigen and antibodies were negative, while *Scedosporium* sp. was detected in the sputum culture. Despite no history of bronchial asthma and low IgE levels, the diagnosis of ABPM caused by *S. apiospermum* was considered based on clinical criteria [3] (Table 1).

**Table 1:** New clinical diagnostic criteria for ABPM in Japan [3].

1. Current or previous history of asthma or asthmatic symptoms 2. Peripheral blood eosinophilia (≥500 cells/mm^3^) 3. Elevated total serum IgE levels (≥417 IU/mL) 4. Immediate cutaneous hypersensitivity or specific IgE for filamentous fungi 5. Presence of precipitins or specific IgG for filamentous fungi 6. Filamentous fungal growth in sputum cultures or bronchial lavage fluid 7. Presence of fungal hyphae in bronchial mucus plugs 8. Central bronchiectasis on CT 9. Presence of mucus plugs in central bronchi, based on CT/bronchoscopy or mucus plug expectoration history 10. High attenuation mucus in the bronchi on CT

Filamentous fungi in criteria 4 to 6 should be identical.

Patients that meet 6 or more of these criteria are diagnosed with ABPM.

**Fig. 3:**
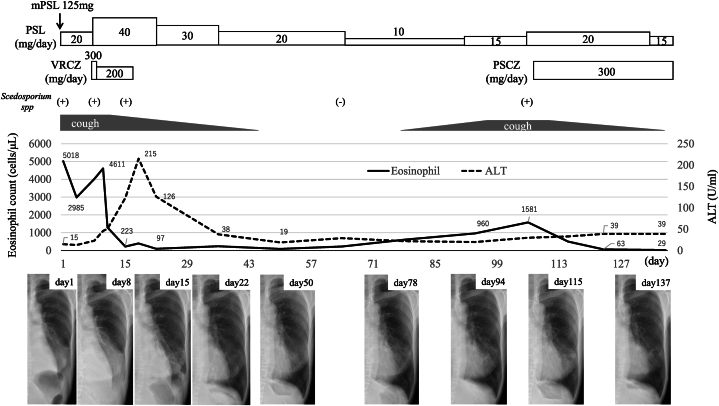
Clinical course of the case. Arrow indicates methylprednisolone (mPSL). Bars indicate the duration of prednisolone (PSL) and antifungal treatment (VRCZ, voriconazole; PSCZ, posaconazole), and arrows correspond to the isolation of *Scedosporium* sp. in sputum cultures. The presence (+) or absence (−) of *Scedosporium* sp. isolation in sputum culture.

Prednisolone at 20 mg/day (0.6 mg/kg/day) initially failed for 1 week; thus, it increased to 40 mg/day alongside voriconazole (VRCZ), yielding symptom, eosinophil count, and imaging findings (Fig. 3). Prednisolone was then tapered every 2 weeks, and every 4 weeks after reaching 20 mg/day. VRCZ ceased after a week due to hepatic impairment, with worsening liver function (AST 78 U/L, ALT 128 U/L, ALP 102 U/L, γ-GTP 87 U/L, LD 270 U/L), where ALT was more than three times the normal value. Liver function improved after discontinuing VRCZ and administering ursodeoxycholic acid. Prednisolone alone worsened symptoms at 10 mg/day, with increased cough, eosinophilia, and lung shadows. *Scedosporium* sp. reappeared in the sputum, worsening ABPM. Prednisolone was increased to 20 mg/day, and improvement followed with the posaconazole (PSCZ), guided by drug susceptibility testing (Table 2) and European Global Guidelines [6]. After 4 weeks at 20 mg/day, the prednisolone dose was tapered, without ABPM recurrence.

**Table 2:** MICs of antifungal drugs against the *S. apiospermum* isolate.

Antifungal drug	MIC (mg/L)
Micafungin	>16
Caspofungin	8
Amphotericin B	4
Itraconazole	>8
Voriconazole	2

## 3 Discussion

In this case, two points emerged: First, allergic bronchopulmonary mycosis (ABPM) associated with the rare fungus *S. apiospermum* is extremely uncommon. Second, Japan's new clinical diagnostic criteria for ABPM enable diagnosis in patients without concurrent bronchial asthma or low serum IgE levels, typical in non-*Aspergillus* ABPM.

*Scedosporium* sp. belong to Phylum Ascomycota, Order Microascales, Family Microascaceae, which are saprophytic fungi widely distributed in nature, including soil, marshes, and sludge [6,7]. In pathological specimens, fungal hyphae are identifiable through PAS, Gomori methenamine silver (GMS), and Grocott staining. Differentiating them from *Aspergillus* can be difficult, with morphological identification post-cultivation being most useful. These fungi can cause opportunistic infections in patients with neutropenia due to chemotherapy for hematologic malignancies, hematopoietic stem cell transplantation, or immunosuppressive drug use [6,8].

*S. apiospermum* primarily infects through inhalating conidia or local skin invasion [9]. Clinical symptoms vary, including respiratory, eye, central nervous system, soft tissue, endocarditis, and disseminated infections [5]. Mortality is high post-infection. Prognosis depends on factors beyond antifungal choice. *Scedosporium* sp.*,* akin to *Aspergillus,* are more invasive with higher mortality rates.

In a 2015 Japanese survey, *Aspergillus* caused 88 % of ABPM cases, with limited data on other organisms. Besides *Aspergillus*, *Schizophyllum commune, Candida albicans*, *Penicillium*, and *Mucor* were common. According to Chowdhary et al., who summarized case reports, the top three fungal species in non-*Aspergillus* ABPM cases were *C. albicans*, *Bipolaris*, and *S. commune*, accounting for 84 % of cases [10]. Fungal species identification, especially non-*Aspergillus* is challenging, often requiring genetic testing or specialized consultation. ABPM cases associated with *Scedosporium* sp. are extremely rare, with only a few case reports available [11]. Genetic testing was conducted at the Chiba University Medical Mycology Center to identify *Scedosporium* sp. The internal transcribed spacer (ITS) region of fungal ribosomal DNA (rDNA) is highly variable and essential for species identification through PCR analysis. The ITS region's species-specific sequences, located between the 18S, 5.8S, and 28S rRNA genes, makes it valuable for identifying fungi at the species level [12]. However, facilities capable of ITS gene analysis are limited. Gene analysis using ITS sequences showed consistency between *S. apiospermum* detected in sputum cultures and the genetic analysis of mucous plug pathology samples from the lesion, confirming the causative fungal species. This level of reporting, including both culture results and genetic analysis of the mucous plug in the lesion, is unprecedented.

Historically, diagnosing ABPM, caused by fungi besides *Aspergillus,* relied on Rosenberg's and the International Society for Human and Animal Mycology (ISHAM)'s criteria [1,2]. However, there are cases where diagnosis is challenging, such as those without a history of bronchial asthma or cystic fibrosis, cases with low serum IgE levels, and cases where evaluating fungus-specific IgE, precipitating antibodies, or immediate hypersensitivity reactions is difficult. Symptoms like wheezing, cough, and dyspnea are common, but ABPM from non-*Aspergillus* sp. often occurs without coexisting asthma, unlike ABPA. Chowdhary et al. reported that 32 % of patients with non-*Aspergillus* ABPM had a history of bronchial asthma [10] and in a study of 15 cases of ABPM caused by *S. commune* in Japan, eight cases (53 %) were found to have coexisting bronchial asthma [13]. When clinically diagnosing ABPA/ABPM, the sensitivities of the new diagnostic criteria, Rosenberg criteria, and ISHAM criteria were reported to be 49.2 %, 82.7 %, and 94.4 %, respectively, with the new diagnostic criteria showing the highest sensitivity [3]. Diagnosing non-*Aspergillus* ABPM is challenging due to the difficulty in detecting specific IgE and precipitating antibodies. Future development of allergy tests for non-*Aspergillus* sp. is needed.

First-line treatment for ABPM involves systemic corticosteroids and oral azole antifungal drugs [14,15]. Corticosteroids directly induce apoptosis in eosinophils and suppress type 2 immune responses and IgE production by acting on Th2 cells. Regarding *S. commune*, which is frequently encountered among non-*Aspergillus* fungi causing ABPM [16], several case reports have suggested the usefulness of administering azole antifungal drugs or combining them with corticosteroids [17,18]. Both oral corticosteroids and antifungal drugs frequently cause relapse upon dose reduction or discontinuation, necessitating long-term administration. During long-term administration, attention must be given to chronic lower respiratory tract infections or osteoporosis from steroid use, and the risk of the emergence of azole-resistant fungi.

In Japan, the treatment guidelines for *S. apiospermum* are currently being developed by the Japanese Society for Medical Mycology. The European Confederation of Medical Mycology has proposed global guidelines in collaboration with ISHAM [6]. These global guidelines strongly recommend voriconazole as the first-line treatment for all patterns of organ involvement. Amphotericin B formulations are not recommended because of repeated in vitro resistance and breakthrough infections. As second-line options, itraconazole, isavuconazole, and posaconazole are recommended, although evidence is limited. In this case, voriconazole was discontinued because of hepatic impairment; however, antifungal treatment was resumed because of the recurrence of ABPM with prednisolone alone. Because the drug sensitivity test results showed resistance to itraconazole, improvement was achieved using posaconazole, following global guidelines. Thus, unlike *Aspergillus* sp., the sensitivity to antifungal agents varies for *Scedosporium* sp., making species identification crucial. As respiratory specialists, we suspected ABPM based on clinical symptoms, tests, and imaging, and performed bronchoscopy. However, mycology expert input was crucial for identifying the fungus. In rare mycosis cases, specialized support is often needed for accurate identification and antifungal testing, making collaboration essential. This teamwork enabled us to identify the fungus and develop an effective treatment plan.

As this is a single case report, the generalizability of the results is therefore limited. Knowledge about diagnosing and treating ABPM caused by non-*Aspergillus* fungi, particularly *Scedosporium* sp., is limited, requiring more case accumulation. Patients may also respond differently to treatment side effects and effectiveness. Large-scale clinical studies and multicenter collaborations to study non-*Aspergillus* ABPM are needed. Improving diagnostic techniques and developing new treatments are essential to enhance diagnostic accuracy and optimize treatment outcomes.

## 4 Conclusions

Here, we present a rare case of ABPM caused by *S. apiospermum*. Such cases, not linked to *Aspergillus,* often occurs in patients without asthma, diagnosed using updated criteria. Genetic analysis aids in identifying fungal species, guiding effective antifungal medications and improving outcomes. Different sensitivities underscore the importance of identifying fungal species.

## Funding

There has been no financial support for this work.

## Ethical approval

This study was conducted in accordance with the principles of the Declaration of Helsinki. The national “Ethical Guidelines for Life Sciences and Medical Research involving Human Subjects” do not require an ethics review committee for case reports. We posted information about case reports to patients who visited our hospital and gave them the opportunity to refuse a case report if they did not agree. The authors declare that appropriate written informed consent was obtained to publish this case report and accompanying images.

## CRediT authorship contribution statement

**Ryota Okazaki:** Writing – original draft. **Shino Arita:** Writing – review & editing. **Hinako Hoshio:** Writing – review & editing. **Naoki Uetani:** Writing – review & editing. **Genki Inui:** Writing – review & editing. **Hiroki Ishikawa:** Writing – review & editing. **Takafumi Nonaka:** Writing – review & editing. **Hiroki Kohno:** Writing – review & editing. **Yoshihiro Funaki:** Writing – review & editing. **Tomoya Harada:** Writing – review & editing. **Masato Morita:** Writing – review & editing. **Akira Watanabe:** Writing – review & editing. **Akira Yamasaki:** Writing – review & editing.

## Declaration of competing interest

The authors have no conflicts of interest to declare.
